# Crohn’s Disease Diagnosed Intraoperatively During a Trauma Exploratory Laparotomy

**DOI:** 10.1155/crgm/1839198

**Published:** 2025-12-30

**Authors:** Jacob Surma, Danielle Hebert, Nathanial Riggan, William Cirocco, Akram Alashari

**Affiliations:** ^1^ Department of Surgery, Covenant Healthcare College of Medicine, Central Michigan University, Mount Pleasant, Michigan, USA, cmich.edu

**Keywords:** Crohn’s disease, exploratory laparotomy, incidental finding, inflammatory bowel disease, trauma surgery

## Abstract

**Background:**

Crohn’s disease, a chronic inflammatory bowel disease, commonly presents with abdominal pain, diarrhea, and weight loss, typically leading to diagnosis through endoscopic or imaging studies. Its incidental discovery during emergent trauma surgery is rare.

**Case Presentation:**

We report the case of a 25‐year‐old male who sustained blunt abdominal trauma in a dirt‐bike accident, presenting to the emergency department the same day with progressive abdominal pain. Emergent exploratory laparotomy for a suspected bowel perforation revealed characteristic “creeping fat,” inflammatory adhesions, and an enterocolonic fistula, suggesting undiagnosed Crohn’s disease. The presence of acute inflammation necessitated extended small bowel resection and precluded primary anastomosis. Postoperatively, the patient developed recurrent intra‐abdominal abscesses, including a subhepatic collection measuring 5.7 × 2.6 × 5 cm, as well as pleural effusions, necessitating multiple drainage procedures, targeted antibiotics, and nutritional optimization. A multidisciplinary team—including trauma surgeons, gastroenterologists, infectious disease specialists, and interventional radiologists—collaborated to manage both acute injuries and the newly recognized chronic inflammatory condition. Final pathology revealed multifocal colonic mucosal ulceration with acute inflammation, ileocolic fistula formation, and colonic perforation, consistent with a penetrating (fistulizing) phenotype, ileocolonic Crohn’s disease.

**Conclusion:**

This case illustrates the importance of maintaining a broad diagnostic perspective during trauma surgery and highlights how an unrecognized inflammatory bowel disease can complicate postoperative recovery. Early suspicion and tailored interventions are crucial for mitigating complications such as abscess formation and sepsis. A prompt gastroenterology consult and confirmatory investigations are recommended to guide long‐term management of Crohn’s disease. Ultimately, this report underscores that vigilance for atypical findings in emergent settings can significantly improve patient outcomes.

## 1. Introduction

Crohn’s disease is a form of inflammatory bowel disease (IBD) characterized by chronic, relapsing inflammation of the gastrointestinal (GI) tract. It has an estimated prevalence of 319 to 721 per 100,000 in North America and Europe, with a rising global incidence [1, 2]. Typically manifesting in young adults, Crohn’s disease commonly affects the terminal ileum and colon but can involve any part of the GI tract [3]. Patients often present with abdominal pain, diarrhea, weight loss, and fatigue, prompting diagnostic evaluations through endoscopy, imaging, and histopathology [4]. While most patients present with characteristic symptoms, asymptomatic cases do occur, with incidental diagnosis reported in approximately 2% of patients in large registry studies [5]. The implications of discovering Crohn’s disease during acute trauma surgery, however, remain poorly characterized in the literature.

Exploratory laparotomy is a standard surgical intervention in acute abdominal trauma when there is concern for underlying visceral injury, hemorrhage, or hemodynamic instability, facilitating direct inspection of the internal organs and enabling control of hemorrhage and contamination [6]. While incidental findings during trauma surgery can occur, the discovery of chronic diseases like Crohn’s disease is rarely documented. Recent emergency department trauma cohorts show that incidental findings on CT are common and frequently underdocumented. In a two‐center cross‐sectional study, 25% of chest and 32% of abdominopelvic CTs in trauma patients contained incidental findings, over half clinically significant, yet documentation occurred in only 31%–44% of cases.

There are few reported cases of Crohn’s disease being incidentally discovered during emergent surgery for traumatic injuries, making this case particularly noteworthy [7]. The present case involves the incidental discovery of Crohn’s disease during an exploratory laparotomy conducted after a dirt‐bike accident. Although the patient had experienced recurrent abdominal pain, he lacked an established IBD diagnosis, making this an unexpected finding in the trauma setting. The presence of Crohn’s disease required immediate adaptation of surgical techniques to address the increased risk of complications due to the fragile and acutely inflamed bowel. Active inflammation not only complicates surgical decision‐making but also increases the potential for postoperative complications such as anastomotic leaks or infections [8].

This report highlights the importance of considering underlying chronic conditions during trauma evaluations and demonstrates how such findings can influence surgical decision‐making in the acute setting. By sharing this unique case, we contribute to the growing body of knowledge regarding the intersection of chronic disease and trauma surgery, offering insights that may be valuable for both trauma and GI surgeons.

## 2. Case Presentation

A 25‐year‐old male sustained blunt abdominal trauma in a high‐impact dirt‐bike accident in the early afternoon. He presented to the emergency department the same evening with progressive abdominal pain that had worsened to 8/10 severity over several hours since the accident. Additional injuries included right shoulder and forearm abrasions and bruising. Preoperative CT imaging demonstrated inflammatory changes involving the terminal ileum, right hemicolon, and transverse colon with mesenteric combing, suggestive of Crohn’s disease. Emergency exploratory laparotomy was performed the following day for pneumoperitoneum and clinical peritonitis. The patient had no established diagnosis of IBD, but retrospective questioning revealed recurrent abdominal pain previously attributed to “gastric ulcers,” despite the absence of prior endoscopic evaluation.

The primary traumatic injury was a perforated hepatic flexure of the colon with extensive fecal peritonitis, evidenced by approximately 1500 cc of contaminated fluid evacuated from the abdominal cavity. Intraoperatively, the surgeon noted “creeping fat” and dense adhesions characteristic of Crohn’s disease.

The intraoperative findings of suspected Crohn’s disease significantly influenced surgical decision‐making. Beyond the traumatic perforations at the hepatic flexure, the surgeon encountered an enterocolonic fistula approximately 50 cm proximal to the ileocecal valve, where serosa‐to‐serosa adhesions between the small bowel and mid‐ascending colon suggested chronic inflammatory changes rather than acute trauma. This finding necessitated an extended small bowel resection of approximately 50 cm to ensure adequate margins around the inflammatory process. Most critically, the presence of acute inflammation in the remaining transverse colon precluded primary anastomosis, a decision that likely avoided anastomotic complications in the setting of active IBD. The surgeon opted for an end ileostomy instead, recognizing that the inflamed bowel was unsuitable for immediate reconstruction. Additionally, two large‐bore intra‐abdominal drains were placed given the increased risk of leak and abscess formation in the setting of both fecal contamination and underlying IBD.

In summary, the operative management included exploratory laparotomy, right hemicolectomy, end ileostomy, partial omentectomy, partial enterectomy, and placement of negative pressure wound therapy. The fascia was closed primarily with PDS suture; however, due to the contaminated nature of the case (wound classification #4 from extensive fecal peritonitis), the subcutaneous tissue and skin were intentionally left open and managed with negative pressure wound therapy to prevent superficial surgical site infection while allowing fascial healing. In the postoperative period, he was initiated on intravenous methylprednisolone (60 mg IV daily) for suspected active inflammation related to Crohn’s disease and empiric intravenous metronidazole (500 mg q6h) with levofloxacin (750 mg q24 h) for broad intra‐abdominal anaerobic and Gram‐negative coverage. He stabilized clinically and was discharged home once he tolerated an oral diet and demonstrated appropriate ileostomy output.

Approximately ten days after the initial surgery (September 4th), the patient was readmitted for planned delayed primary closure of his midline laparotomy wound due to complications with the wound VAC from multiple wound clots and bleeding. The operative findings demonstrated excellent granulation tissue without fluid collection, and closure was performed successfully. During this admission, the infectious disease team was consulted and prescribed ceftriaxone, continued metronidazole, and added fluconazole, though the specific indication for this consultation is not clearly documented in the available records. The patient also developed pneumonia and pleural effusions, which were managed conservatively by pulmonology. Following clinical improvement and nutritional optimization with total parenteral nutrition, he was discharged on September 7th with oral ciprofloxacin (500 mg BID), amoxicillin–clavulanate (875/125 mg BID), and fluconazole (200 mg daily) for 10 days.

Four days after discharge (September 11th), he was readmitted with recurrent pleural effusions and subsequently transferred to our facility for interventional radiology evaluation, approximately 18 days after the initial trauma.

**Figure 1:**
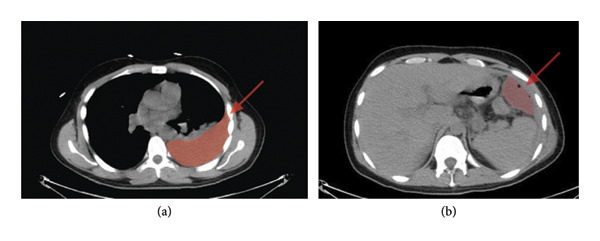
(a) Left‐sided pleural effusion and (b) LUQ complex fluid collection.

On arrival at our facility, his abdomen was tender without peritoneal signs, and his vitals were within normal limits except for tachypnea. Laboratory evaluation revealed leukocytosis (WBC 15.4 × 10^3^/μL) and lactic acidosis (2.5 mmol/L). CT scan demonstrated a moderate left pleural effusion and a large left upper quadrant fluid collection measuring 7.9 × 4.4 × 8.4 cm, consistent with an abscess (Figure 1).

He was admitted to the acute care and trauma surgery service. Under CT guidance, interventional radiology placed a drain into the LUQ fluid collection on September 12th, yielding approximately 10 mL of purulent fluid. A left‐sided thoracentesis performed on September 13th yielded 700 mL of cloudy, amber‐colored pleural fluid. The patient was started on ampicillin–sulbactam IV for continued broad‐spectrum coverage. Nutritional optimization was prioritized due to the evidence of malnutrition (prealbumin: 10.0 mg/dL).

**Figure 2:**
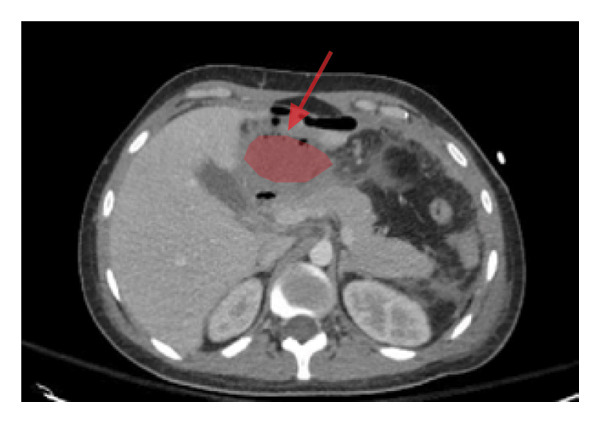
Subhepatic abscess.

One week after admission to our facility (September 18th), the patient’s midline wound developed cellulitis and a superficial abscess, which was drained at the bedside with expression of copious purulent fluid. Persistent leukocytosis prompted repeat CT imaging that revealed a new subhepatic abscess measuring 5.7 × 2.6 × 5 cm which was not amenable to IR drainage (Figure 2). In the context of recurrent abscesses and culture‐proven *E. coli*, his antibiotic regimen was escalated to meropenem (2 g IV q8h) for maximal Gram‐negative and anaerobic coverage. With continued antibiotics, drainage, and supportive care, the patient’s condition gradually improved, and he was eventually discharged home.

Final surgical pathology revealed multifocal colonic mucosal ulceration with acute inflammation, ileocolic fistula formation, and colonic perforation, consistent with Crohn’s ileocolitis. Following resolution of acute complications, the patient was scheduled for gastroenterology consultation to discuss long‐term management of his newly diagnosed Crohn’s disease.

## 3. Discussion

Incidental discovery of Crohn’s disease during trauma laparotomy is rarely documented, though isolated case reports exist [7]. In this case, intraoperative recognition of chronic IBD directly influenced surgical decision‐making in three key ways. First, an extended small bowel resection of approximately 50 cm was performed to address an enterocolonic fistula and ensure adequate margins around inflammatory disease—well beyond what would be required for the traumatic perforation alone. Second, primary anastomosis was deemed unsafe due to acute inflammation at the transverse colon landing site, and an end ileostomy was created instead. Third, large‐bore intra‐abdominal drains were placed given the heightened risk of leak and abscess formation.

The decision to avoid primary anastomosis was supported by the literature on anastomotic leak rates. In trauma settings, primary repair after colonic perforation carries a leak rate of approximately 4%–5% [9], while ileocolic anastomosis in Crohn’s disease resections shows similar rates [10]. However, the combination of active inflammation, fecal contamination, and underlying chronic disease in this case created compounded risk. Anastomotic leaks are associated with 10%–20% increased mortality [11], making damage control principles paramount. Current guidelines for traumatic colon injuries recommend diversion when significant comorbidities are present [12], a principle appropriately applied here. By creating an end ileostomy rather than attempting primary reconstruction in inflamed, contaminated tissue, the surgical team likely prevented catastrophic anastomotic complications.

Preoperative imaging and clinical history provided clues to underlying Crohn’s disease that were not recognized prior to surgery. CT demonstrated terminal ileum inflammation and mesenteric combing. Additionally, the patient reported chronic abdominal pain previously attributed to “gastric ulcers” without endoscopic evaluation. While the emergent trauma setting justifiably prioritized immediate life‐threatening injuries, these findings reveal a missed diagnostic opportunity that could have influenced preoperative risk stratification.

The intersection of Crohn’s disease and abdominal trauma has particular clinical significance. Recent literature demonstrates that patients with IBD have increased susceptibility to bowel perforation even with minor abdominal trauma [13–15]. Chronic inflammation and fibrosis weaken the bowel wall, making it more vulnerable to mechanical disruption. This case illustrates this well, where what might have produced only contusion in healthy bowel resulted in perforation in the setting of undiagnosed Crohn’s disease. The presence of an enterocolonic fistula further suggests that chronic inflammatory changes preceded the traumatic event.

Reflecting upon this, revisiting preoperative imaging and symptom history once patients are stabilized is an important step that may reduce complications following the acute setting. Patients such as this one warrant gastroenterology consultation and follow‐up, even when acute injuries are the primary concern. Early recognition is critical given that patients with Crohn’s disease require specialized postoperative management, including consideration of early medical therapy to prevent disease recurrence after resection [16]. In this case, prompt gastroenterology involvement could have facilitated earlier initiation of appropriate medical therapy once acute surgical complications resolved.

The patient’s postoperative course was complicated by multiple intra‐abdominal abscesses, pleural effusions, and a prolonged infectious course requiring escalating antimicrobial therapy and repeated drainage procedures. While these complications are consistent with fecal peritonitis, as sepsis, pulmonary complications, and abscess formation are well‐documented sequelae [17], the role of underlying Crohn’s disease and perioperative management decisions in this outcome warrants examination.

A notable management decision was the initiation of intravenous methylprednisolone (60 mg daily) immediately postoperatively for suspected active Crohn’s inflammation. The rationale for this intervention is unclear from available documentation, particularly given that the literature examining perioperative corticosteroid use in IBD patients undergoing surgery demonstrates significantly increased postoperative complication rates. Literature examining preoperative corticosteroid use in IBD patients undergoing surgery demonstrates significantly increased postoperative complication rates, with doses exceeding 40 mg daily associated with particularly high risk [18]. The mechanism appears to involve immunosuppression and blunted inflammatory responses rather than impaired wound healing, with corticosteroids reducing proinflammatory cytokines necessary for infection control [19, 20]. It is difficult to determine whether the early steroid administration in this case contributed to the infectious complications, though the temporal relationship and severity of abscess formation raise concern.

This case highlights a clinical dilemma without clear evidence‐based guidance regarding the management of newly discovered IBD in the setting of contaminated emergency surgery. The competing priorities of controlling potential IBD‐related inflammation versus avoiding immunosuppression in an infected field represent a difficult balancing act. Without detailed documentation of abscess locations relative to surgical anastomoses or staple lines, we cannot definitively attribute complications to Crohn’s‐specific factors versus consequences of fecal peritonitis. Our experience suggests that when definitive surgical management is achieved through resection of diseased bowel, delaying immunosuppressive therapy until resolution of acute postoperative complications may be prudent. During the immediate postoperative period, supportive measures including bowel rest and parenteral nutrition can reduce intestinal inflammation without requiring immunosuppression. Initiation of IBD‐directed medical therapy can be reconsidered once the patient has recovered from immediate postoperative complications.

As stated previously, long‐term gastroenterology follow‐up is essential once Crohn’s disease has been identified. The American Gastroenterological Association provides comprehensive guidelines for managing Crohn’s disease after surgical resection; however, clear evidence‐based tools for managing newly diagnosed IBD in the acute trauma setting remain limited [21]. In such cases, integration of established principles for managing intra‐abdominal infections becomes critical. Adequate source control, appropriate antimicrobial therapy, and nutritional optimization are foundational to managing the complex interplay of trauma, contamination, and chronic inflammatory disease [22].

This case required coordination among multiple specialties, including gastroenterology for IBD management, infectious disease for antimicrobial stewardship and abscess management, interventional radiology for drainage procedures, and nutrition services for optimization of healing. The complexity of managing concurrent acute trauma complications and newly recognized chronic disease exceeds the scope of surgical care alone. Early involvement of these services facilitated timely interventions and likely improved outcomes in this challenging clinical scenario. Prompt consultation of gastroenterology, infectious disease, and nutritional support is recommended when IBD is discovered in the acute surgical setting.

To summarize, we present important lessons for managing unexpected findings during trauma surgery. When surgeons encounter intraoperative signs like creeping fat, inflammatory adhesions, or abnormal bowel appearance, IBD should be recognized as a possibility. In these situations, surgical planning needs to shift toward damage control. This includes resecting diseased segments with adequate margins, avoiding anastomosis when tissue is inflamed or contaminated, placing drains appropriately, and accepting that reconstruction may need to happen at a later operation.

Beyond the operating room, there are systems‐level changes that could prevent similar diagnostic delays. Once patients are stabilized from their acute injuries, their preoperative imaging should be reviewed for findings that may have been missed during initial trauma evaluation. Additionally, ensuring gastroenterology consultation before discharge is important for confirming the diagnosis and setting up appropriate long‐term management of IBD.

There are, however, important limitations. A single case cannot establish the best approach for managing newly discovered Crohn’s disease in the trauma setting. We lacked complete documentation from the outside hospital, which limited our ability to determine exact abscess locations or understand all the reasoning behind certain management choices. It remains unclear whether the complications stemmed primarily from the Crohn’s disease itself, from the fecal contamination, or from the decision to start steroids immediately after the surgery. Despite these uncertainties, this case demonstrates that staying alert to chronic disease findings during trauma surgery can meaningfully impact surgical decisions and patient care.

## 4. Conclusion

This case emphasizes the rare but clinically significant possibility of uncovering undiagnosed Crohn’s disease in the acute setting of trauma laparotomy. While management typically focuses on controlling life‐threatening injuries, surgeons should remain alert to intraoperative findings suggestive of chronic inflammatory disorders. Recognizing these subtleties can shape operative decisions, reduce postoperative complications, and ensure prompt initiation of long‐term medical management. Close collaboration with gastroenterology and other specialty services is essential for optimizing patient outcomes, particularly given the complex interplay between underlying Crohn’s disease and acute traumatic injuries. Patients diagnosed with Crohn’s disease in this context require careful, lifelong follow‐up to prevent recurrence and optimize quality of life. This underscores the necessity of considering chronic disease processes even when the immediate focus is on acute traumatic injuries.

## Consent

Written informed consent was obtained from the patient. He agreed for publication of this case report as well as imaging. A copy of this consent form is available upon request.

## Conflicts of Interest

The authors declare no conflicts of interest.

## Author Contributions

All authors contributed equally to the creation of the manuscript, imaging review, and revisions.

## Funding

No funding was received for this publication.

## Data Availability

Data sharing is not applicable to this article as no datasets were generated or analyzed during the current study.
